# The profile of general practitioners (GPs) who publish in selected family practice journals

**DOI:** 10.1186/1756-0500-4-164

**Published:** 2011-05-26

**Authors:** J Soler-González, C Ruiz, C Serna, JR Marsal

**Affiliations:** 1 GREDELL Research Group. Regional Primary Care Management Office, IDIAP Jordi Gol, Catalan Institute of Health, University of Lleida, Lleida, Spain

## Abstract

**Background:**

Providing support for research is one of the key issues in the ongoing attempts to improve Primary Care. However, when patient care takes up a significant part of a GP's time, conducting research is difficult. In this study we examine the working conditions and profile of GPs who publish in three leading medical journals and propose possible remedial policy actions.

**Findings:**

The authors of all articles published in 2006 and 2007 in three international Family Medicine journals - Annals of Family Medicine, Family Practice, and Journal of Family Practice - were contacted by E-mail. They were asked to complete a questionnaire investigating the following variables: availability of specific time for research, time devoted to research, number of patients attended, and university affiliation. Only GPs were included in the study. Three hundred and ten relevant articles published between 2006 and 2007 were identified and the authors contacted using a survey tool. 124 researchers responded to our questionnaire; 45% of respondents who were not GPs were excluded. On average GPs spent 2.52 days per week and 6.9 hours per day on patient care, seeing 45 patients per week. Seventy-five per cent of GPs had specific time assigned to research, on average 13 hours per week; 79% were affiliated to a university and 69% held teaching positions.

**Conclusions:**

Most GPs who publish original articles in leading journals have time specifically assigned to research as part of their normal working schedule. They see a relatively small number of patients. Improving the working conditions of family physicians who intend to investigate is likely to lead to better research results.

## Background

The promotion of quality research contributes to a successful Primary Care service. However, when family physicians spend most of their time attending patients, their opportunities for conducting research are limited.

Research in Primary Care has three positive effects: patients receive improved quality of care; physicians' job satisfaction and motivation increase; and the health system benefits from improved clinical efficacy and effectiveness [1-3].

For many years, family physicians have sought to increase funding and to extend the time available for research that would aid public health and university teaching [4]. GPs need answers to clinical questions from studies related to family medicine [5].

Several studies have investigated the factors associated with positive attitudes towards research among physicians [6], and have identified possession of a higher scientific degree, teaching and research experience, and perceived practical application as the most influential. Moreover, publishing allows physicians to share knowledge and to promote evidence-based medicine and best practices [7].

In this study we intend to establish whether researchers who publish in international journals devote all their time to patient care or whether part of their schedule is set aside for research. In so doing, we aim to define the profile and working conditions of the GPs who publish in leading family practice journals.

## Methods

The research protocol of the pilot study was approved by the Institutional Review Board of the Jordi Gol i Gurina Primary Care Research Center in Barcelona, Spain. All the corresponding authors of articles published in 2006 and 2007 in three Family Medicine journals - Annals of Family Medicine (AFM), Family Practice (FP), and Journal of Family Practice (JFP), with impact factors of 3.8, 1.55 and 1.27 respectively - were contacted by E-mail. Journal selection was not based on a systematic search but on criteria such as high impact factor and indexation in Pubmed.

Our sample comprises authors who published articles in "family medicine journals". These are defined as journals with "family medicine", "family practice," or "family physician" in their titles, and indexed in Pubmed. We also stipulated that the author's E-mail address had to be included.

Seven indexed journals contained the descriptive term *FAM *in the title. Four had to be excluded: *American Family Physician*, because it does not include original articles, *Canadian Family Physician *and *The Journal of the American Board of Family Medicine*, because they contain only domestic articles, and *Family Medicine*, because it does not include the authors' E-mail addresses in the abstract.

All non-original articles published in the three journals selected were also excluded.

All the corresponding authors of the articles published during the period of study were contacted and invited to complete a questionnaire investigating the following items: the number of days per week and hours per day spent attending patients, the availability of paid time for research, time devoted to research, and the number of patients seen per week. Participants were also asked whether they had any academic affiliation with a university or engaged in any other kind of teaching. All corresponding authors were included in the study. Non-GPs were excluded.

### Statistical analysis

ANOVA was performed for the three journals and the three countries/groups of countries with the highest number of publications (the US, the UK, and Others). The other countries (Israel, Greece, Canada, Estonia, Germany, Australia, Ireland and Belgium) were grouped together for the analysis because they only produced a single publication each. The three cases of authors who published on two different occasions were classified as new. We used a t-test to assess the statistical significance.

## Results

Three hundred and ten original papers were published between 2006 and 2007 in the three selected journals. Replies were received from 124 authors (40%). Of these replies, 45.2% were rejected because they were written by physicians other than GPs (internists, psychiatrists, psychologists, etc.). There were no differences in the response rate between GPs who published in AFM and FP (56%), but this rate was substantially lower among the authors who published in JFP (16.7%) (Table 1).

**Table 1:** Percentages of participating GPs according to journal

	Impact factor of the journal	Articles published	Authors who replied n (%)	GPs (% of replies)
Annals of Family Medicine	3.8	105	46 (43.8%)	26 (56.5%)

Family Practice	1.55	187	72 (38.5%)	41 (56.9%)

Journal of Family Practice	1.27	18	6 (33.3%)	1 (16.7%)

The GPs attended patients for an average of 2.5 days per week (SD 1.38), for 7 hours (SD 2.8) per day, and saw 45 patients per week (SD 30). Seventy-five per cent were allocated time for research (mean 13.8 hours per week), 79.4% were affiliated to a university, and 69.1% were involved in teaching. Authors who published in AFM devoted fewer days to patient care (p = 0.11), and saw fewer patients (p = 0.005) (Table 2).

**Table 2:** GP profile according to journal

	AFP		Family Practice		JFP		p-value**
	
	N	Mean (CI95%)	N	Mean (CI95%)	N	Mean	
Days per week attending patients	26	2.3 [1.66 - 2.84]	41	2.7 [2.36 - 3.13]	1	1	0.114

Hours per day attending patients	26	6.3 [5.18 - 7.35]	41	7.4 [6.57 - 8.26]	1	8	0.056

Number of patients attended per week	26	33.1 [23.52 - 42.63]	41	54 [44.56 - 63.54]	1	15	0.005

Hours dedicated to research	26	16.9 [11.49 - 22.28]	41	11.4 [8.75 - 14.05]	1	32	0.211

Minutes spent attending each patient	24	33.1 [24.63 - 41.62]	41	26.2 [20.14 - 32.26]	1	32	0.067

Proportion of time dedicated to research (%)	25	47 [33.97 - 60.05]	41	35.6 [28.98 - 42.29]	1	80	0.124

**: Tested between AFP and Family Practice (JFP excluded).

The authors from the US and the UK (the most prolific) devoted fewer days to patient care and saw fewer patients, but did not spend less time attending patients on workdays. They all had university affiliations (Table 3).

**Table 3:** Profile of the GPs with the highest number of publications (UK, US and Others).

	UK	US	Others	p-value
GPS with time scheduled for research (%)	81.25	90.91	60	**0.0266**

Days per week attending patients	1.88	2.34	3.02	**0.0135**

Hours per day attending patients	7.94	6.32	6.97	**0.0916**

Number of patients attended per week	40.44	29.64	59.73	**0.0033**

Hours dedicated to research	16.13	20.23	7.85	**0.0009**

Minutes spent attending each patient	21.36	35.51	28.05	**0.0307**

Proportion of time dedicated to research	49.76	54.66	24.74	**0.0001**

University affiliation (%)	93.75	90.91	60	**0.0053**

Proportion of GPs who teach (%)	50	81.82	70	**0.1122**

## Discussion

Analyzing the profile of GPs who publish their research can help design interventions able to stimulate general evidence-based research in the future. The main finding of this study is that GPs who publish in prestigious journals do not see patients every day; most of these doctors have time specifically set aside for research and are university-affiliated.

Interestingly, these physicians continue to see more than 40 patients a week. This indicates that they are clinically active, often at the price of seeing too many patients on the days they practice, although seeing more patients per day and devoting entire days to scientific research may in fact be an effective way of optimizing the time they spend with patients. Unfortunately, the majority of family doctors devote considerably more hours per week to patient care: According to Wensing [8], GPs in 10 European countries spent a mean of 41.3 hours (SD 12.2) per week on patient care, a figure that is substantially higher than the one we found in the authors with high publishing rates. Breaking the figures down by countries, Wensing [8] found that GPs worked an average of 53 hours per week in Belgium, 41 hours in Denmark and Switzerland, 39 hours in The Netherlands, 37 hours in Spain, 34 hours in Germany and Slovenia, 31 hours in Norway, and finally 28 hours in the UK.

These differences in the availability of time highlight the importance of raising the profile of Primary Care in the scientific environment. Observers of the discipline agree that family medicine does not contribute enough to research and note that increasing financial and other pressures within its academic departments are making research more difficult [9].

The profile of our GPs from the countries with low publication rates is no different from that of their counterparts in the US or the UK. However, in terms of university affiliation, physicians in the US and the UK are at an advantage because the discipline of Family Medicine appears to be better established there.

Today, the time factor is critically important for a GP aiming to achieve professional excellence. Salmon et al. found that it is not "time, money, and lack of relevance" that dissuade practitioners from taking part in research but a lack of prioritization based on perceived threats to their professional autonomy and a lack of personal incentive [10]. Time management is a major factor in GP practice, one that underlies a wide range of processes and decisions [11]. In most countries a GP will earn substantially more by doing clinical work than by doing research.

The characteristics that were positively associated with initiating research in a study of practitioners in the UK [1] included involvement in teaching, having research-active partners, the availability of protected time, and working in a large practice. The most commonly perceived barriers were lack of time (92%), lack of staff to collect data (73%), and insufficient funding (71%).

In our study many of the GPs who published the most were affiliated to universities. We believe that doctors who are involved in teaching at the University are more motivated to carry out research and have more time to do so. As in other disciplines, the vast majority of family medicine research is carried out in academic departments. This suggests that the discipline should strategically focus its research and development efforts on the academic environment. Academic family medicine is now represented in most medical schools, and international family medicine journals receive excellent papers from all over the world [12]. In fact, as many as 79% of the authors who publish in leading family practice journals are attached to universities.

The methods that different countries have applied to develop family medicine research vary, but they usually include a combination of international support, local enthusiasm, willingness to change, and some luck [13].

### Limitations

We were unable to use all the journals devoted to Family Medicine. The reasons were the lack of original articles, the presence of studies by GPs from highly circumscribed regions, the influence of regional differences, and country affiliation, since journals are probably more likely to publish studies by researchers discussing locally relevant healthcare problems than those written by foreign GPs.

We consider the response rate to be high (40%) and the data obtained from our respondents showed the expected trend. As we stated in the methods section, our study centered on family physicians, and all other medical specialists were excluded. In all cases we wrote to the corresponding authors, because they are usually the leaders of research projects and take responsibility for publication. The absolute number of responses we used was enough to make it comparable with other studies and the results were consistent with the referenced literature. Due to the time commitment needed for research, it is more practical for a GP to be a member of a research team rather than the chief investigator and/or first author of a publication. This means that the study may have captured only a subgroup of publishing GPs and that there may be a risk of an answering bias.

We do not have data on the amount of clinical work done by GPs who do not publish academic work. Judgements about the relative clinical load of GPs who publish are therefore speculative. Many studies by GPs are published in non-indexed journals. These studies are obviously relevant to an assessment of the research conducted in primary care but they are not easy to locate [14]. In a broad discipline like family medicine, researchers are more likely to publish in journals outside the field: For example, half of the research published between 1961 and 2005 in the seemingly focused field of nephrology appeared in non-renal journals [15].

Some of the best primary care research has been published in general medical journals. A single study in a leading general medical journal might be a greater contribution to the discipline than several in a family medicine journal, and would have been missed by the method we used for journal selection. However, we believe that the inclusion of these high impact journals would not have significantly altered the professional profile of the researchers recorded in our study.

## Conclusions

In this study we found that GPs who publish original articles in leading family practice journals have time specifically assigned to research as part of their normal working commitments and see relatively few patients per week. Many of them are also affiliated to universities. Primary care is first contact, continuous, comprehensive, and coordinated care for individuals and populations undifferentiated by age, gender, disease, or organ system [16].

Primary health care research is directly relevant to policy and practice [17]. This is shown by the considerable impact of research projects on processes and policies in health care, knowledge production, and the construction of research capacity among teams [18,19].

Research impact comes through complexities and therefore unpredictable social processes [20,21]. The researchers should take partnerships and collaborations seriously, to ensure their topic is relevant to current policies and practices, and disseminate their work actively and skillfully, using networks to raise awareness of the findings [22]. The development of research practices allows individual primary care teams to become more involved in research at a variety levels [19].

Therefore, in order to promote quality medical research and teaching in Primary Care, we must seek strategies to allocate time for research during the normal working day. Further studies should explore ways of enabling more family doctors to perform research. For example, in 1998, the RCGP developed a pilot scheme to accredit UK general practices undertaking primary care research and development. The Assessment Schedule included two levels: a Collaborative Research Practice with little direct experience of gaining project or infrastructure funding; or an Established Research Practice with more experience of research funding and activity and a sound infrastructure to allow for growth in capacity. The process for assessment involved the review of written documentation and an assessment visit by a multidisciplinary team. In 2001, recommendations were made to launch the scheme, renamed Primary Care Research Team Assessment (PCRTA), formally. The role of primary care research networks has been highlighted in relation to support and mentoring for research practices undergoing assessment. The new assessment scheme will help primary care trusts and individual practices to prepare and demonstrate their approach to research governance in a systematic way [23].

High profile researchers who lead competitive projects are well acquainted with the research world, but many GPs would be keen to participate if they received encouragement and support [24]A key challenge for university-based departments is the decision to stay focused on clinical general practice or to develop wider expertise in health service research. The quality of the research versus teaching load also needs to be carefully considered. Will there only be a few research-based departments and will they have to have a full quota of expertise in qualitative methods, epidemiology, economics, and statistics to be successful in gaining grants from the prestigious research charities? Arguments of critical mass come into play here. Does this mean integrating primary care research with health services research and public health? This is becoming a global problem. The tension here is between research that is generalizable and of international stature and the development of a wider base of research-ready clinicians [19].

A possible solution would be to allow interested doctors to participate in research on a part-time basis and to provide them with institutional support and resources [25,26]. The history of research in general practice is a progression from exceptional individuals to multidisciplinary academic departments that balance the need to keep in touch with the grassroots of clinical practice whilst competing in academic environments. Although essentially still fighting for their recognition within university settings, these departments are also witnessing internal mid-life problems. Teaching and research are struggling to remain bed-fellows, as community-based teaching becomes a major commitment and as researchers are driven to publish and increase research quality. Success also brings other challenges. Generalists, by their nature, turn their hands to many things, often with great enthusiasm. The discipline paradoxically encompasses multidisciplinary primary care using an ever-increasing range of research methods. As the academic world adapts to the fragmented face of primary care delivery in newly organized health care contexts, a sense of questioning the meaning of it all is occasionally voiced, and as in most mid-life situations, it seems there are significant changes ahead [27,28].

Further studies are needed to define more precisely the mechanisms for increasing GP involvement in academic activities. Research on primary care provides the means to improve the organization of services and to question beliefs or behaviours. The generalist clinician stands at one of the most complex intersections in society, as science, represented by psychological, physiological, and pharmaceutical interventions, and the humanities, as represented by the social contexts, beliefs, narratives, and shared mythologies, intermingle in consultations to formulate, on the one hand, interventions and their resultant outcomes and, on the other, extended stories that serve to embellish lives with hopes and fears. It is no wonder that research in primary care has, in distinction to other realms of medicine that largely embrace positivistic perspectives, uses the most diverse set of research methods [19].

## Competing interests

The authors declare that they have no competing interests.

## Authors' contributions

JSG designed the study, participated in the planning of the study, wrote the first draft of the manuscript and was responsible for study coordination. CR and CS supervised the study including its design, and participated in the writing of the manuscript. JRM performed the statistical analysis, and participated in the writing of the manuscript. All authors read and approved the final manuscript.
